# Identification, Characterization and Expression of *Methuselah-Like* Genes in *Dastarcus helophoroides* (Coleoptera: Bothrideridae)

**DOI:** 10.3390/genes7100091

**Published:** 2016-10-21

**Authors:** Zhengqing Zhang, Huapeng Wang, Chunfeng Hao, Wei Zhang, Miaomiao Yang, Yong Chang, Menglou Li

**Affiliations:** 1 Laboratory of Forestry Pests Biological Control, College of Forestry, Northwest Agriculture and Forestry University, Yangling 712100, China; zhangzhengqing0802@hotmail.com (Z.Z.); hppwang@163.com (H.W.); hlzsdau2009@163.com (C.H.); zhangwei1062002@163.com (W.Z.); changyongzzzx@163.com (Y.C.); 2 Institute of Biotechnology, Shaanxi Xueqian Normal University, Xi’an 710100, China; yangmmgg@163.com

**Keywords:** *Dastarcus helophoroides*, *mth-like* genes, expression profile, development, lifespan, reproduction, stress resistance

## Abstract

*Dastarcus helophoroides*, which has a relatively longer lifespan compared to other insects, is one of the most effective natural enemies of many large-body long-horned beetles. Methuselah (Mth) is associated with the lifespan, stress resistance, and reproduction in *Drosophila melanogaster*, but Mth is not present in non-drosophiline insects. A number of *methuselah-like* genes (*mth-likes*, *mthls*) have been identified in non-drosophiline insects, but it is still unknown whether they are present in *Dastarcus helophoroides*. We identified three novel *mth-like* genes in *D. helophoroides*: *mth-like1*, *mth-like2*, and *mth-like5*, and carried out bioinformatic analysis based on the full-length nucleic acid sequences and deduced amino acid sequences. Real-time quantitative polymerase chain reaction (RT-qPCR) showed variations in expression patterns of *mth-like* genes in different tissues (highly expressed in reproductive systems) and at different developmental stages, indicating that *mth-likes* were likely be involved in reproduction and development. The altered mRNA expression in aging adults and under oxidation, high temperature, and starvation stress, indicated that *mth-like* genes were likely to be involved in aging and the resistance of oxidation, high temperature, and starvation. These results characterize, for the first time, the basic properties of three *mth-like* genes from *D. helophoroides* that probably play important roles in development, aging, reproduction, and stress resistance.

## 1. Introduction

Methuselah (Mth), a G protein-coupled receptor (GPCR), was originally identified in *Drosophila melanogaster*. In *D. melanogaster*, *mth* mutation has been shown to extend the lifespan by 35%, and increase resistance to starvation, high temperatures, and paraquat treatment [1]. GPCRs are a large and important group of receptor proteins involved in signal transduction. They are classified into five large families, namely glutamate-like receptors, rhodopsin-like receptors, adhesion, frizzled, and secretin-like receptors [2]. Mth is a subfamily of the secretin-like family (family B) that has been reported as being insect-specific [3]. Mth shows an N-terminal Mth ectodomain and a C-terminal seven transmembrane (7tm) domain [4,5,6,7]. Family B of the GPCRs is a small, but structurally and functionally diverse, group of proteins that includes receptors for polypeptide hormones, molecules thought to mediate intercellular interactions at the plasma membrane and a group of *Drosophila* proteins that regulate stress responses and longevity [6]. Like other class B GPCRs, the large (195 residue) amino-terminal extracellular domain of Mth is essential for ligand-binding [6]. However, the sequence similarity of the *mth* gene to other class B GPCRs is observed solely within the transmembrane regions [1]. The crystal structure shows that the Mth ectodomain consists primarily of β-sheets [5], revealing a topology distinct from that of other hormone receptors [8,9].

However, *mth* mutation has an offsetting negative effect on fertility in *D. melanogaster*, which demonstrates the trade-off effect between longevity and fertility [10,11]. Complete knock-out of *mth* has been reported to result in lethality of the embryo, which illustrates the crucial function of Mth receptors to maintain normal growth and development [12,13,14]. It has also been reported that Mth receptors were involved in the regulation of the male germline stem cell population and the sensorimotor functions in adult *Drosophila* [15,16]. The Mth receptor also has the function of strengthening the *Drosophila* neural synaptic transmission efficiency. Previous study has demonstrated that mutations in the *mth* gene reduced evoked neurotransmitter release by ~50%, and decreased synaptic area and the density of docked and clustered vesicles [12]. As research continues, more physiological functions of Mth receptors will gradually be found.

In addition to *mth*, a large number of *methuselah-likes* (*mth-likes*, *mthls*) have been suggested as being present in insects. So far, fifteen *mth-like* genes from *D. melanogaster*, seven from *Anopheles gambiae*, four from *Bombyx mori*, four from *Apis mellifera*, three from *Acyrthosiphon pisum*, and five from *Tribolium castaneum* have been identified [4,17,18,19,20]. *Mth* and *mth-likes* are believed to have arisen from one ancestral gene and to have been duplicated in insects [20], but it is unknown whether they share similar functions in insects.

*Dastarcus helophoroides* (Faimaire) (Coleoptera: Bothrideridae) is one of the most effective natural enemies of many large-body long-horned beetles, including *Anoplophora glabripennis*, *Monochamus alternatus*, *Batocera horsfieldi*, and *Massicus raddei* [21]. *D. helophoroides* larvae are ecto-parasitoids of late instar larvae, pupae, and young adults of long-horned beetles, which makes it a potential biological control agent for pest management [22,23,24]. In addition, *D. helophoroides* is also a suitable insect for aging studies due to the long lifespan of adults. Under laboratory conditions, *D. helophoroides* can live for more than eight years with continued sexual reproduction [25], providing a unique resource for molecular and physiological studies of development and reproduction. Although the morphology and physiology of *D. helophoroides* have been widely studied, the underlying molecular mechanism of development and reproduction remain unknown [26].

Thanks to sequencing and annotation of the *D. helophoroides* transcriptome using Illumina HiSeq 2000 (Beijing Genomics Institute, BGI, Shenzhen, Guangdong, China) sequencing technologies, genes involved in development, aging, reproduction, and resistance to adversity can be easily identified. The aim of this study, therefore, is to identify, characterize, and determine the expression profiles of *mth-like* genes in *D. helophoroides*. Our expression analyses of *mth-likes* in different tissues, at different life stages, in adults differing in age, and in adults under the stress of oxidation, high temperature, and starvation, could provide the basis for further study about their possible functions in *D. helophoroides* and other insects.

## 2. Materials and Methods

### 2.1. Insects

The larvae, pupae, and adults of *D. helophoroides* were obtained from a laboratory colony (the laboratory of Forestry Pests Biological Control, College of Forestry, Northwest Agriculture and Forestry University, Yangling, China), and were maintained in a temperature-controlled room at 23 ± 1 °C, 70%–80% relative humidity (RH) with a photoperiod cycle of 16 hL/8 hD [25,27]. The larvae of *D. helophoroides* were reared in test tubes using the alternative host, the pupae of *Tenebrio molitor*, while adults were reared in plastic boxes and fed on an artificial diet that predominantly consisted of silkworm pupa powder, sugar, yolk, agar, and water [26].

### 2.2. Identification and Rapid Amplification of cDNA Ends

The *mth-like* genes were searched using the *D. melanogaster mth* sequence as a query in the sequences from the adult *D. helophoroides* transcriptome database (previously published under the accession GBCX00000000) that had already been identified and annotated by Blastx [28] against the non-redundant (NR) protein database at the National Center for Biotechnology Information (NCBI) with a cut-off E-value of 1.0 × 10^−5^ [27,29]. Based on the cDNA fragments obtained by searching the transcriptome annotation files, gene-specific primers were designed using Primer Premier 5.0 (Premier Biosoft International, Palo Alto, CA, USA) to amplify the full length *mth-likes* of *D. helophoroides*. The primers used for PCR-based cloning are shown in Table 1. The rapid amplification of cDNA ends PCR (RACE-PCR) technique was applied to obtain the full-length *mth-like* cDNAs with a SMARTer™ RACE cDNA Amplification Kit (Clontech, Beijing, China) according to the manufacturer’s instructions. Total RNA isolated from adult insects using a UNIQ-10 Trizol RNA extraction kit (Sangon, Shanghai, China) was used to obtain cDNA templates needed in RACE-PCR. The PCR products were cloned into the pMD-19-T Vector (TaKaRa, Kyoto, Japan) and sequenced by Sangon Biotech (Shanghai, China).

### 2.3. Sequence and Phylogenetic Analysis

Open reading frames (ORF) were identified and translated with online ORF Finder software [30]. The cDNA sequences were compared with the other similar *mth-like* genes registered in GenBank using Blastx (searching the protein database using a translated nucleotide query) [28]. The sequence alignment and identity analysis were carried out using the DNAMAN software package (Lynnon Corporation, Pointe-Claire, QC, Canada) [31]. The molecular weights and isoelectric points of predicted proteins were calculated by the SWISS-PROT (ExPASy server) program “Compute pI/Mw” [32]. The evolutionary relationships within the mth-like family were inferred using the neighbor-joining (NJ) method implemented in MEGA 5 (Center for Evolutionary Medicine and Informatics, Tempe, AZ, USA) with default settings and bootstrap support based on 1000 iterations [33].

### 2.4. Expression Analysis through RT-qPCR

To investigate the expression patterns of *mth-like* transcripts at different life stages larvae of each instar (1st–6th instar), pupae, and newly-emerged adults of both male and female were collected. To investigate tissue-specific expression of *mth-like* transcripts in *D. helophoroides*, the newly-emerged adults were used and the following tissues which cover the whole insect were isolated for real-time quantitative polymerase chain reaction (RT-qPCR) analysis: head, thorax, midgut, hindgut, male reproductive system, female reproductive system, and residual body (mainly body wall and muscles). The tissues were stored in RNA storage solution (CWBIO, Beijing, China) until use in RNA isolation and cDNA synthesis. To explore the expression patterns of *mth-like* transcripts during aging, adults fed with artificial diets were also collected and then classified into eight groups (aged 2, 4, 10, 12, 18, 20, 26, and 30 months) according to the survival time from emergence. Each group contains an equal number of adult females and males (1:1). To analyze the mRNA expression profiles of *mth-like* genes in adult *D. helophoroides* under oxidative stress, males and females (two months old) were collected at specific times (0, 1, 2, 3, 4, 5, 6, 12, 18, and 24 h) after exposure to 20 mM paraquat. To analyze the mRNA expression profiles of *mth-likes* in adult *D. helophoroides* under high temperature stress, males and females (two months old) were collected at specific times (0, 0.5, 1.0, 1.5, 2.0, 3.0, 6.0, and 12.0 h) after exposure to the high temperature of 45 °C. To analyze the mRNA expression profiles of *mth-like* genes in adult *D. helophoroides* under starvation stress, males and females (two months old) were collected at specific times (0, 2, 4, 6, 8, 10, 12, and 14 days) after exposure to the condition without food, but with water. All samples that were collected were frozen in liquid nitrogen and stored at −80 °C.

Total RNA of each sample above was isolated with a UNIQ-10 Column Trizol Total RNA Isolation Kit (Sangon Biotech, Shanghai, China) according to the manufacturer’s protocol. The concentration and quality of total RNA were determined by spectrophotometry using a Maestro-NANO UV spectrophotometer (MaestroGen, Las Vegas, NV, USA). The first-strand cDNA was synthesized with a PrimeScript RTreagent Kit with gDNA Eraser (TaKaRa Bio Inc., Dalian, China) according to the manufacturer’s instructions using 1 μg total RNA in a 20 μL final reaction volume. The cDNA was stored at −20 °C. In accordance with reference gene selection in *D. helophoroides*, EF-1α was chosen as endogenous control in different development stages and tissues; α-tubulin can be used as reference gene in adult *D. helophoroides* for different survival times. For each target gene and reference gene, specific primers were designed with Primer Premier 5.0 software (PREMIER Biosoft International, Palo Alto, CA, USA) (Table 1). Each pair of primers was validated by calculating standard curves with a 5× serial dilution of *D. helophoroides* cDNA as a template.

The expression of *D. helophoroides mth-like* transcripts was assayed by RT-qPCR using a Bio-rad IQ5 Thermol System with SYBR Green Mix (CWBIO, Beijing, China). Cycling conditions were as follows: 95 °C for 3 min, 50 cycles of 95 °C for 30 s, 58 °C for 30 s, 72 °C for 30 s, followed by melting temperature analysis: 65–95 °C held for 10 s for each degree. The relative expression was determined using the 2^−ΔΔCt^ method [34,35]. qPCR was repeated for a total of three biological replicates with three technical replicates each, which included a no template control and a no reverse-transcriptase control.

### 2.5. Statistical Analysis

All data were presented as mean ± SD (standard deviation). Significant differences between each group were analyzed by using Tukey’s test [36]; *p* < 0.05 was considered statistically significant. One-way ANOVA was used for multiple comparisons using SPSS 20.0 (IBM SPSS Statistics, Chicago, IL, USA).

## 3. Results

### 3.1. Identification and Cloning of Putative Mth-Like Genes

To facilitate the identification of *mth-like* transcripts in *D. helophoroides*, we searched the *D. helophoroides* transcriptome database. Three *D. helophoroides* unigenes homologous to insect *mth-like* genes were identified in an assembly. To confirm the validity of the assembled transcripts, each putative *mth-like* was cloned and sequenced. Cloning and sequencing of full length cDNA of these *mth-like* sequences were accomplished using 5′- and 3′-rapid amplification of cDNA ends (RACE). Gene-specific primers designed for each *mth-like* sequence are shown in Table 1.

**Figure 1:**
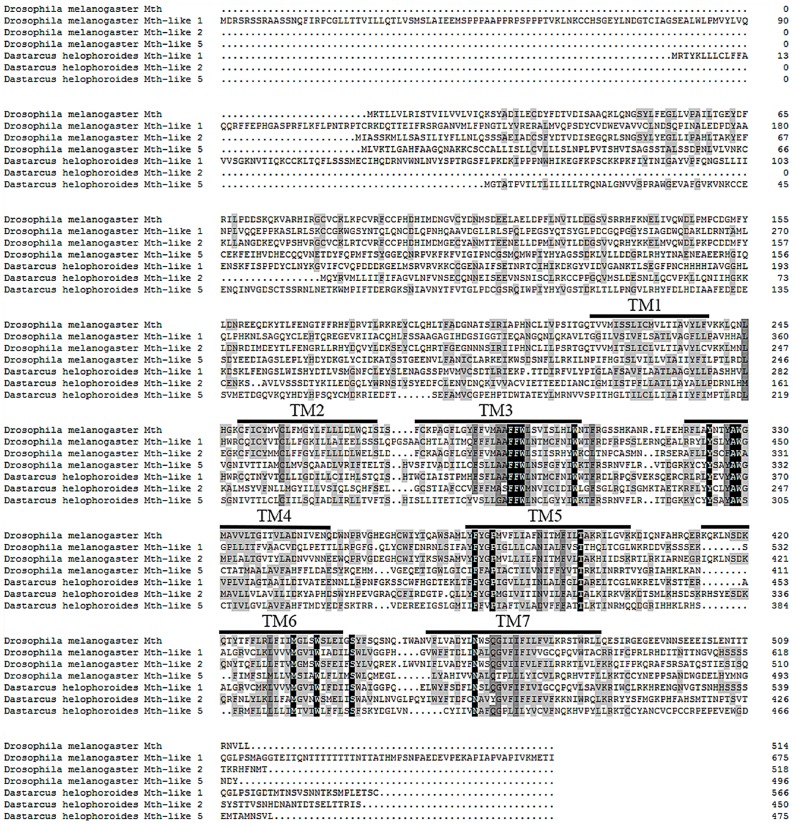
Sequence alignment of Mth/Mth-likes. Sequences (cloned or annotated) are *D. helophoroides* Mth-likes (Mth-like1, Mth-like2, and Mth-like5), *D. melanogaster* Mth (NP-523871.1) and *D. melanogaster* Mth-likes (Mth-like1 NP-573140.1, Mth-like2 NP-788462.2, and Mth-like5 NP-650126.2). TM1–TM7 indicate the transmembrane domains. Identical or similar residues are highlighted black or grey, respectively, in the alignment. ORF: open reading frames.

Three cDNA clones of *mth-like* genes with full-length sequences were obtained. Determination of their putative amino acid sequences with Blastx tools showed that all three are members of the Mth-like (Mthl) family. The three nucleotide sequences were named as *mth-like1*, *mth-like2*, and *mth-like5*, and the corresponding proteins were, therefore, designated as Mth-like1, Mth-like2, and Mth-like5, due to their homology with *Drosophila* and *T. castaneum* proteins. Sequences were deposited in GenBank with accession numbers KM588897, KU363815, and KU363816. The full lengths of *mth-like1*, *mth-like2*, and *mth-like5* were 2600 bp, 1875 bp, and 2312 bp, which contained open reading frames (ORF) of 1701 bp, 1353 bp and, 1428 bp and encoded proteins with 566, 450, and 475 amino acid residues, respectively. The deduced molecular weights were 63.8 kDa, 51.8 kDa, and 54.4 kDa and the isoelectric points (PI) were 8.67, 8.06, and 6.01, respectively (Table 2). All of these putative Mth-likes have conserved seven transmembrane domains, although the moderate degree of sequence similarity of Mth-like1, Mth-like2, and Mth-like5 compared with the Mth (NP-523871.1) in *D. melanogaster* are only 24%, 29%, and 25%, respectively (Figure 1).

### 3.2. Phylogenetic Analysis of Mth-Like Genes

**Figure 2:**
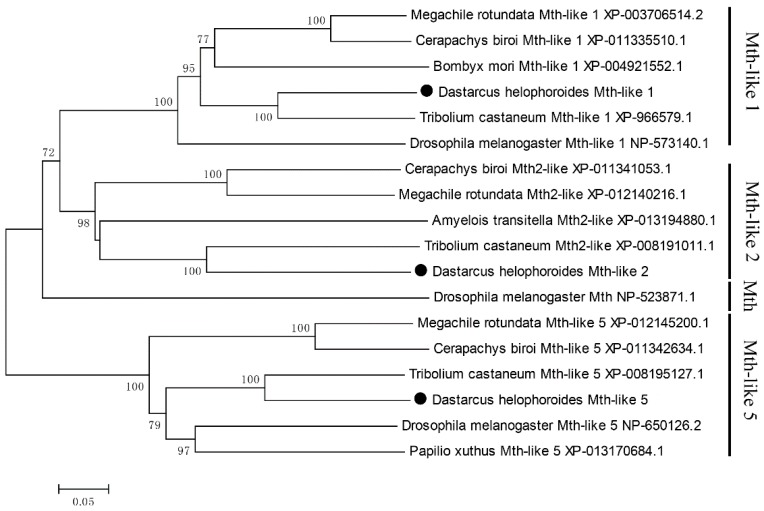
Molecular phylogenetic analysis by the neighbor-joining (NJ) method. Bootstrap values with 1000 trials re-indicated on the branches. GeneBank accession numbers of amino acid sequences used to generate the tree are shown in the Figure. *D. helophoroides* sequences are marked by a black circle.

To assess the relationships amongst the three *D. helophoroides* Mth-like sequences and those identified from other insects, phylogenetic analysis was carried out using the neighbor-joining (NJ) method implemented in MEGA 5 with default settings and bootstrap support based on 1000 iterations (Figure 2). The results showed that the total 18 Mth/Mth-like proteins were divided into four clusters: Mth-like1, Mth-like2, Mth-like5, and Mth. Specifically, *D. helophoroides* Mth-likes (Mth-like1, Mth-like2, and Mth-like5) were more closely related to *T. castaneum* Mth-likes (Mth-like1, Mth-like2, and Mth-like5), showing 64%, 53%, and 66% identity, respectively, with a bootstrap value of 100%.

### 3.3. Developmental Expression Profiles

**Figure 3:**
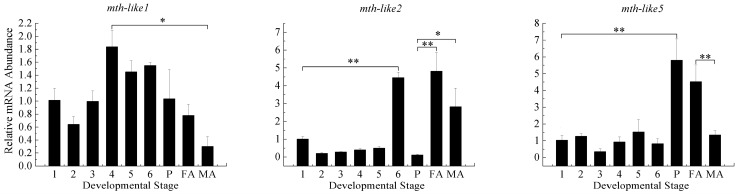
Developmental expression levels of *mth-like* genes. The relative expression indicates the level of each *mth-like* transcript normalized to the internal standard of EF-1α, compared to expression levels of the first instar. Data are shown as the mean ± SD. Asterisks indicate differences that are statistically significant (*, *p* < 0.05; **, *p* < 0.01). 1–6, 1st–6th instar larvae; P, pupae; FA, female adults; MA, male adults.

To better understand the physiological roles of *D. helophoroides mth-like* genes in development, their expression profiles were examined by RT-qPCR at different life stages with EF-1α as a reference gene. In general, *mth-likes* are expressed throughout all the developmental stages examined, but they exhibited different expression patterns (Figure 3). The mRNA levels of *mth-like1* displayed a relatively high expression level at the late larval stage (fourth, fifth, and sixth larval stages) and *mth-like2* were high at the sixth larval and adult stages, while the mRNA levels of *mth-like5* were highly expressed at the pupal and adult stages. The mRNA levels of all the three *mth-like* genes of female adults were significantly higher than that of males. These results showed that the three *mth-like* genes were expressed differently during development indicating they might play different roles in the development of *D. helophoroides*.

### 3.4. Tissue Expression Profiles

**Figure 4:**
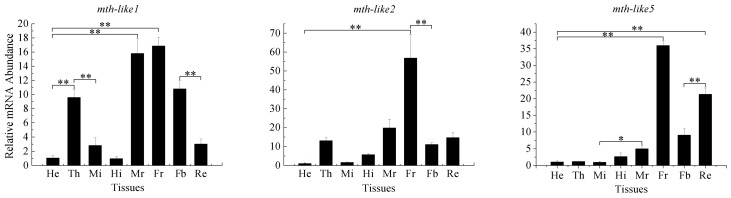
Tissue expression profiles of *mth-like* genes. The relative expression indicates the level of each *mth-like* transcript normalized to the internal standard of EF-1α, compared to the expression level of head. Data are shown as the mean ± SD. Asterisks indicate differences that are statistically significant (*, *p* < 0.05; **, *p* < 0.01). He, head; Th, thorax; Mi, midgut; Hi, hindgut; Mr, male reproductive system; Fr, Female reproductive system; Fb, fat body; Rb, residual body.

To explore tissue distribution, transcript abundance in the head, thorax, male and female reproductive systems, midgut, hindgut, fat body, and residual body were determined by RT-qPCR with EF-1α as a reference gene. In general, although *mth-like* genes could be detectable in all of the examined tissues, they displayed different expression patterns and appeared to be specific (Figure 4). The expression levels of *mth-like1* in the male and female reproductive system (15.80- and 16.86-fold relative to the head, respectively) were significantly higher than those in any other tissues. The expression levels of *mth-like1* in the thorax and fat body (9.58- and 10.79-fold relative to the head, respectively) were significantly higher compared to those of the head, midgut, hindgut, and residual body, but significantly lower than in the male and female reproductive systems. The expression level of *mth-like2* was the highest in the female reproductive system (56.78-fold relative to the head). Similarly, *mth-like5* showed a significantly higher level in the female reproductive system (35.97-fold relative to the head), whilst the expression level in the residual body was significantly higher than in any other tissues (head, thorax, male reproductive systems, midgut, hindgut, and fat body), but lower than in the female reproductive system. These results demonstrated that these three *mth-like* genes were differently expressed in the tissues and displayed high expression levels in the female reproductive system.

### 3.5. Expression Profiles during Aging Adults

**Figure 5:**
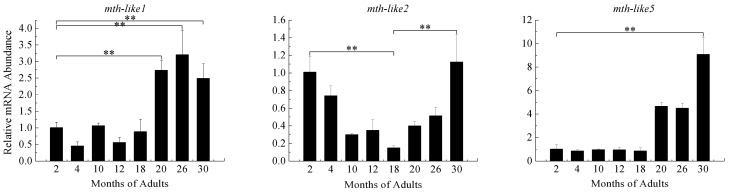
Expression of *mth-like* genes in different groups of adults (aged 2, 4, 10, 12, 18, 20, 26, and 30 months). The relative expression indicates the level of each *mth-like* transcript normalized to the internal standard of α-tubulin, compared to expression level of the first group (two-months). Data are shown as the mean ± SD. Asterisks indicate differences that are statistically significant (*, *p* < 0.05).

To explore the possible roles of the *mth-like* genes in aging, expression levels of these three *mth-like* genes in different age groups of adults were determined by RT-qPCR with α-tubulin as a reference gene. As shown in Figure 5, the expression level of *mth-like1* increased significantly in the older groups (aged 20, 26, and 30 months), increased 2.74-fold for 20 months, 3.21-fold for 26 months, and 2.49-fold for 30 months, relative to the two-month group. The expression level of *mth-like2* decreased from the two-month group, reaching the lowest point (15% of the two-month group) in the 18-month group, and then increased in the older groups (aged 20, 26, and 30 months), reaching a relatively high level (1.12-fold relative to the two-month group) in the 30-month group. The expression of *mth-like5* remained relatively steady in the young groups (aged 2, 4, 10, 12, and 18 months) and then significantly increased in the older groups (aged 20, 26, and 30 months), reaching the highest level (8.78-fold relative to the two-month group) in the 30-month group. Altogether, the expression levels of *mth-like1* and *mth-like5* were significantly increased in older groups (aged 20, 26, and 30 months) and *mth-like5* displayed a more remarkable increase (4.28- to 8.78-fold) than *mth-like1* (2.49- to 3.21-fold). Meanwhile, the expression level of *mth-like2* began changing from the youngest adults (two-months) and recovered in the 30-month group.

### 3.6. Expression Profiles under the Oxidative Stress

**Figure 6:**
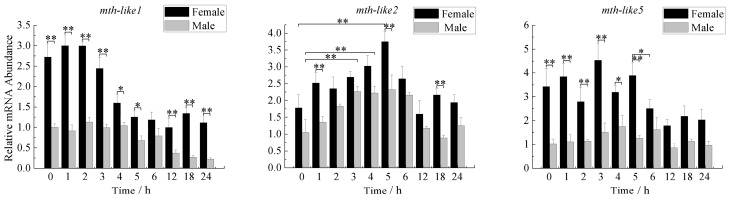
Expression profiles under oxidative stress. The relative expression indicates the level of each *mth-like* transcript normalized to the internal standard of α-tubulin, compared to expression levels of the first group (0 h). 0–24 h means different time under the oxidative stress. Data are shown as the mean ± SD. Asterisks indicate differences that are statistically significant (*, *p* < 0.05; **, *p* < 0.01).

To determine whether *D. helophoroides mth-like* genes respond to oxidative stress, newly-emerged adults were exposed to paraquat and an abundance of these three *mth-like* transcripts were determined over time after exposure by RT-qPCR with α-tubulin as a reference gene. As shown in Figure 6, the expression levels of *mth-like1* in both male and female adults significantly down-regulated at the late stage (4–24 h for females, 12–24 h for males) of exposure, decreasing to 36.59%–58.62% of 0-h adults for females, and 22.36%–36.88% of 0-h adults for males. *Mth-like1* expression levels in females were significantly higher when compared with males. On the whole, the expression levels of *mth-like2* in females were higher than that in males but this was not very significant. In female adults, the expression level of *mth-like2* in female adults increased at the early stage of exposure, reaching the highest level (2.11-fold relative to the 0-h group) at five hours before decreasing. While the expression in male adults displayed a relatively stable and high level (2.07–2.23-fold relative to the 0-h group) at the middle stage (3–6 h) of exposure, the expression level fluctuated and was higher than in males, but not very significantly. The expression of *mth-like5* of females fluctuates in the early stage (0–5 h) of exposure and then was slightly down-regulated at the late stage (6–24 h). Furthermore, there is no significant variation in the expression level for males between all of the groups, but a higher expression level was found in females compared to males.

### 3.7. Expression Profiles under the Stress of High Temperature

**Figure 7:**
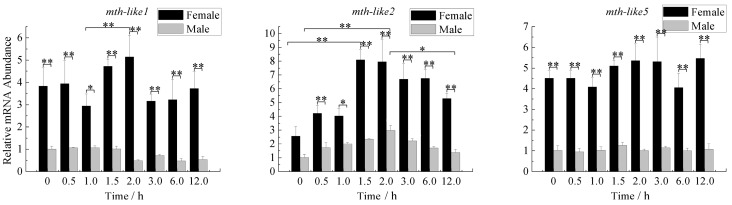
Expression profiles under the stress of high temperature. The relative expression indicates the level of each *mth-like* transcript normalized to the internal standard of α–tubulin, compared to the expression level of the first group (0 h). 0–12 h means different time of adults exposed at a high temperature of 45 °C. Data are shown as the mean ± SD. Asterisks indicate differences that are statistically significant (*, *p* < 0.05; **, *p* < 0.01).

To determine whether *D. helophoroides mth-like* genes respond to high temperature stress, newly-emerged adults were exposed to a high temperature of 45 °C, and then the abundance of *mth-like* genes were detected both in males and females over time after eclosion with α-tubulin as a reference gene. As shown in Figure 7, there is no obvious fluctuation in the expression level of the three *mth-like* genes in either female or male adults with the exposure time increasing, while these three genes exhibited a relatively higher expression level in females than males. For *mth-like1* in females over time, the expression of the gene was slightly increased at middle stages (1.5–2.0 h) compared to that at 1.0 h. Simultaneously, it exhibited a slight decrease in males at the late exposure stage (2.0–12.5 h). The expression of *mth-like2* was significantly up-regulated in female adults (increased more than three-fold compared with the adults at 0-h), which was found at the middle stage (1.5–2.0 h) of exposure. Then, the expression decreased slowly as time went on at the late stage (3.0–12.0 h) of exposure, reaching the lowest level (2.06-fold relative to the 0-h group) at 12.0 h. Meanwhile, the expression of males increased at the early stage (0–2.0 h) and then decreased at the late stage (2.0–12.0 h) and peaked at 2.0 h (2.93-fold relative to the 0-h group). Intriguingly, neither females nor males showed a significant variation in the expression level of *mth-like5* among any of the groups.

### 3.8. Expression Profiles under the Stress of Starvation

**Figure 8:**
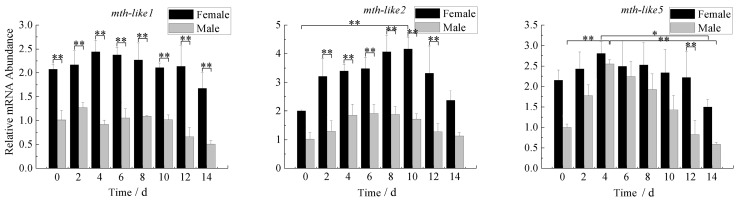
Expression profiles under the stress of starvation. The relative expression indicates the level of each *mth-like* transcript normalized to the internal standard of α–tubulin, compared to the expression level of the first group (0 day). 0–14 days means the starving time of adults. Data are shown as the mean ± SD. Asterisks indicate differences that are statistically significant (*, *p* < 0.05; **, *p* < 0.01).

To determine whether *D. helophoroides mth-like* genes expression changed in response to food deprivation, newly-emerged adults (male and female) were cultured under the condition of no food and abundance of these three *mth-like* genes transcripts was determined by RT-qPCR over time with α-tubulin as a reference gene. As shown in Figure 8, the expression level of *mth-like1* in both females and males showed no obvious regular fluctuation as time went on after exposure. However, the expression in females was significantly higher than in males. The expression of *mth-like2* of females increased at the early stage (0–10 days), reaching the highest level (2.05-fold relative to the 0-day group) 10 days after exposure, and then decreased to the normal level at 14 days (1.01-fold relative to 0 day) after exposure. In addition, the expression of *mth-like2* in males shared a similar trend with that of females, but it peaked (1.91-fold relative to the 0-day group) six days after exposure and then returned to normal (1.12-fold relative to the 0-day group) 14 days after exposure. The expression level in females was significantly higher than in males after exposure. The expression of *mth-like5* in both females and males increased from the early stage (0–4 days) to the highest level at (1.30-fold relative to the 0-day group for females, and 2.55-fold relative to the 0-day group for males, respectively) four days after exposure, and then it decreased with time to the bottom at 14 days, which showed a 30.37% and 41.35% reduction compared with the original levels (0-day adults) for female and male adults, respectively. The expression in females was higher than that of males at early and late stages, but not significantly at the early stage (0–10 days) after exposure.

## 4. Discussion

In this study we identified and characterized three *mth-like* genes from *D. melanogaster* for the first time. The three genes were designated as *mth-like1*, *mth-like2*, and *mth-like5*, and the corresponding proteins were designated as Mth-like1, Mth-like2, and Mth-like5, respectively. Previous studies have demonstrated that *D. melanogaster* Mth-likes (homologous receptors of Mth) show high similarity to Mth and contain similar seven transmembrane (7tm) domains [3,37]. In our study, these three putative Mth-likes consisted of seven conserved transmembrane domains, which is consistent with the finding of a recent study in *T. castaneum* [38], although they only shared a moderate degree of sequence identity (24%, 29%, and 25% for Mth-like1, Mth-like2, and Mth-like5, respectively) with the Mth (NP-523871.1) of *D. melanogaster*.

Phylogenetic analysis suggested that *D. helophoroides* Mth-likes (Mth-like1, Mth-like2, and Mth-like5) were clustered into Mth-like1, Mth-like2, and Mth-like5 groups, respectively, and are very close to *T. castaneum* Mth-likes (Mth-like1, Mth-like2, and Mth-like5) in the tree, showing 64%, 53%, and 66% identity, respectively. A recent study on the expression pattern and phylogenetic relationship of some *mth-likes/mth* in *D. melanogaster* and *T. castaneum* suggested subfunctionalization and acquisition of novel functionalities [3], so the functional divergence most likely occurred between *D. melanogaster mth* and *D. helophoroides mth-like* genes, while *D. helophoroides* Mth-likes were likely to have similar functions with *T. castaneum* Mth-likes. Recent research has revealed that the *Mth/Mthl* gene family is ancient, and *Mthl1*, *Mthl5*, *Mthl14*, and that *Mthl15* are the oldest *Mth/Mthl* gene family paralogs in *Drosophila* [39]. However, it is hard to judge which one is the oldest through phylogenetic analysis in this study, so more detailed analysis and further research is needed.

A diversity in expression patterns for these three *mth-like* genes from *D. helophoroides* was also found in this study. The expression of *mth-like1* displayed a relative high level at the late larval stage (fourth, fifth, and sixth larval stages), *mth-like2* at the sixth larval and adult stages, and *mth-like5* at the pupal and adult stages (Figure 3). Previous studies in *D. melanogaster* have shown that *mth* was expressed in both embryos and third-instar central nervous system (CNS) or discs, *mth-like1* and *mth-like5* were expressed in the embryo, while *mth-like2* was not detected in whole embryos or third-instar brain and imaginal discs using in situ hybridization techniques [3]. A recent study suggested that the mRNA levels of *mth-like1* of *T. castaneum* were highly expressed at the late egg, pupa, and early adult stage, and *mth-like2* and *mth-like5* were highly expressed at the early embryonic and late pupal stages [38]. Similarly to *T. castaneum mth-like* genes, *mth-like* genes of *D. helophoroides* were expressed throughout all of the larval, pupal, and adult stages, but to some extent they display different expression patterns, indicating that *mth-like* genes (*mth-like2* and *mth-like5*) of *D. helophoroides* were likely to have similar functions involved in larval and pupal development and the process of eclosion to *T. castaneum mth-like* genes, while functional divergence most likely occurred between *T. castaneum* and *D. helophoroides mth-like* genes (*mth-like1*).

In addition to development, *mth-like1* and *mth-like2* are also involved in reproduction in *T. castaneum*. In the same recent study, when the expression of *mth-like* genes (*mth-like1* and *mth-like2*) was inhibited, one pair of beetles laid less eggs per day, but *mth-like5* was not involved in fertility [38]. Previous studies have shown that the fertility of *mth*-mutated flies is decreased between 25 °C and 29 °C [11]. In our study, the mRNA levels of *mth-like1*, *mth-like2*, *and mth-like5* were all highly expressed in the female reproductive system (Figure 4). Tissue expression profiles of *mth-like* genes demonstrate that *mth-like* genes are likely to be involved in reproduction in *D. helophoroides* and high expression levels of *mth-like* genes are likely to be crucial for normal fertility. Meanwhile, further research on *mth-like* genes in *D. helophoroides* is needed to explore whether they are functionally consistent in reproduction with *T. castaneum mth-like* genes.

Existing studies have demonstrated that both mutation and suppression of *mth* can enhance the longevity of *D. melanogaster* [1,39]. Conversely, the *T. castaneum* groups with suppression of *mth-like1*, *mth-like2*, and *mth-like5* had a significantly shorter lifespan, which applied to both sexes [38], indicating that both *mth* and *mth-like* genes are involved in insect aging and are functionally divergent. However, both *mth* mutation in *D. melanogaster* and *mth-like* gene suppression in *T. castaneum* have been reported to result in lethality for pre-adults [1,38], which demonstrates that at least some activity of the *mth* and *mth-like* genes are essential for survival. In our study, the expression levels of both *mth-like1* and *mth-like5* increased significantly in the older groups (aged 20, 26, and 30 months), while *mth-like2* firstly decreased, and then increased, finally recovering to the normal level (Figure 5). This may demonstrate that *mth-like* genes are involved in aging and are likely to have positive effects on lifespan of *D. helophoroides*. To some extent, the normal or overexpression of *mth-like* genes is likely to be the reason why *D. helophoroides* adults have a long lifespan.

Reduced signaling via Methuselah (Mth), a G-protein coupled receptor (GPCR) required for neurosecretion, has previously been reported to enhance stress resistance in flies [1,40]. A previous study on *mth-like* genes of *T. castaneum* showed that suppression of *mth-like* genes decreased resistance to the stress of oxidation (*mth-like5*), high temperature (*mth-like1* and *mth-like2*), and starvation (*mth-like1*, *mth-like2*, *mth-like3*, *mth-like4*, and *mth-like5*) [38]. In our study, the mRNA levels of *mth-like1*, *mth-like2*, and *mth-like5* were all responsive to the stress of paraquat (Figure 6), only *mth-like2* was responsive to the stress of high temperature (Figure 7), and *mth-like2* and *mth-like5* were responsive to starvation stress (Figure 8). This demonstrates that both *mth* and *mth-like* genes are likely to be involved in stress resistance and functional divergence may have occurred among insect *mth* and *mth-like* genes. A recent study on the expression pattern and phylogenetic relationship of some *mth*/*mth-like* genes in *D. melanogaster* and *T. castaneum* suggested subfunctionalization and acquisition of novel functionalities [3].

Both reduced expression and overexpression of *mth* targeted to the insulin producing cells (IPCs) of the fly brain were able to extend life and enhance oxidative stress resistance due to Mth’s interaction with β-arrestin, which uncouples GPCRs from their G-proteins [1]. Enhanced longevity and stress resistance in *Caenorhabditis elegans* and flies are often the result of signals from several pathways integrated and coordinated by activation of the forkhead box O (FOXO) transcription factor [41,42,43,44]. The lifespan and oxidative stress effects of MTH signaling are FOXO-dependent and rely not only on the abundance of Mth itself, but also on the abundance of the β-arrestin scaffold protein, which interacts with Mth to inhibit its signaling in critical cellular targets [1]. In summary, this is consistent with a mechanism by which reduced MTH signaling increases longevity and enhances stress resistance by directly reducing systemic insulin-IGF (insulin-like growth factor) signaling (IIS), as it has previously been shown in worms and mice, as well as flies [45,46,47,48,49]. In the present research the regular changes of *mth-like* genes mRNA levels in *D. helophoroides* adults of different age groups (Figure 5), under the stress of oxidation (Figure 6), high temperature (Figure 7), and starvation (Figure 8), are likely the result of Mth-likes’ interaction with other factors. This demonstrates that *mth-like* genes are likely to be involved in a complex mechanism by which changed Mth-likes’ signaling affects longevity and the ability of stress resistance.

## 5. Conclusions

Three *mth-like* genes were identified from *D. helophoroides*, which showed different transcriptional expression profiles at different developmental stages, in various tissues, and in different age groups of adults, as well as in response to the stress of oxidation, high temperature, and starvation. Our findings provide a theoretical basis for further research in molecular biology analysis of *D. helophoroides*, and establish valuable insights for further investigation into the functions of *mth-likes* in insects in the future.

## Figures and Tables

**Table 1:** Gene-specific primers used for rapid amplification of cDNA ends and real-time quantitative PCR of *Dastarcus helophoroides mth-like* genes.

Oligo Name	Oligo Sequence (5′ to 3′)
**Specific primer for 5′ RACE**	
Mth-like1 5′GSP1	TCACCAATAGATGGAAGTCCCTGAGAGC
Mth-like1 5′GSP2	CGCCGATTGGACCGTAGAAGAATGTAAG
Mth-like2 5′GSP1	CAACAGTTGTGGTGTGCCATCTCTAATAAA
Mth-like2 5′GSP2	CAAGAAGGACAGCCATACCCCAAGCATA
Mth-like5 5′GSP1	TTGGAGAACGACGACAGGAAGAACAGC
Mth-like5 5′GSP2	ACGACCAAGCGTATGCCGAATAGTAGC
**Specific primer for 3′ RACE**	
Mth-like1 3′GSP1	TCTTCTCATCGGCGACATTCTCCTCTGC
Mth-like1 3′GSP2	ATTCTTCTACGGTCCAATCGGCGTTCTT
Mth-like2 3′GSP1	AGTTGGTATCATCCGGAAGTTGGCAGAG
Mth-like2 3′GSP2	CTTTATTAGAGATGGCACACCACAACTG
Mth-like5 3′GSP1	GTGGCTGTTCTTCCTGTCGTCGTTCTC
Mth-like5 3′GSP2	GCTGGGGTCCGTCCAAAGGAGGTGATA
**Specific primer for qPCR**	
Mth-like1 S	GCCAACCACAAGTCCTATCAGCT
Mth-like1 A	CACCAATAGATGGAAGTCCCTGA
Mth-like2 S	TTTGGTTAGGATTCAGCGGACT
Mth-like2 A	GGACAGCCATACCCCAAGCA
Mth-like5 S	ACTATTCGGCATACGCTTGGTC
Mth-like5 A	CAGTGATCCGATTTCTTCCCG
**Reference primer for qPCR**	
α-tubulin S	TCGGTGGTGGTACTGGGTCT
α-tubulin A	ACGGCTGTTGAAACTTGAGGA
EF-1α S	TCCTTCAAATATGCCTGGGTACT
EF-1α A	AAATCTCTGTGTGTCCAGGGGCAT

RACE, rapid amplification of cDNA ends; qPCR, quantitative PCR.

**Table 2:** Basic sequence information of *mth-like* genes in *Dastarcus helophoroides*.

Gene Name	Length (bp)	ORF (bp)	Protein Length (aa)	Isoelectric Point	Molecular Weight (kDa)	Accession Number
*mth-like1*	2600	1701	566	8.67	63.8	KM588897
*mth-like2*	1875	1353	450	8.06	51.8	KU363815
*mth-like5*	2312	1428	475	6.01	54.4	KU363816
